# Supplementary material for: Development and Validation of a Deep Learning Model for Histopathological Slide Analysis in Lung Cancer Diagnosis
Source: Cancers (Basel). 2024 Apr 15;16(8):1506. doi: 10.3390/cancers16081506 (PMC11048051; doi:10.3390/cancers16081506)
Supplement: Supplementary file 1 [file cancers-16-01506-s001.zip › cancers-2927701-supplementary.pdf]

Supplementary files for

Development and Validation of a Deep Learning Model for Histopathological Slide Analysis in Lung Cancer Diagnosis

Alhassan Ali Ahmed, Muhammad Fawi, Agnieszka Brychcy, Mohamed Abouzid, Martin Witt and Elżbieta Kaczmarek

Click on the title to move to the page.

Supplementary File S1. *(Pages 2 to 17: navigate to page 2 to check each figure separately)*

Supplementary Figure S1. *Inputs and outputs for CNN model architecture*

Supplementary Figure S2. *comparing the results of the designed algorithm on the collected slides for this side and images from an open-source repository: the Department of Pathology and Laboratory Medicine at Dartmouth-Hitchcock Medical Center (DHMC) [1].*

Table S1. *Comparing the results of the model by images from Department of Pathology and Laboratory Medicine at Dartmouth-Hitchcock Medical Center (DHMC) [1].*

Image nr	AI decision	Array	Accuracy	Pathologist 1	Pathologist 2	Pathologist 3	Pathologist 4	Pathologist 5	Pathologist 6	Pathologist 7	Pathologist 8	Pathologist 9	Pathologist 10	Ref page	Path Accuracy
**1**	Healthy	<0.001	100%	Healthy	Healthy	Healthy	Healthy	Healthy	Healthy	Healthy	Healthy	Healthy	Healthy	'1'!A1	100%
**2**	Healthy	<0.001	100%	Healthy	Healthy	Healthy	Healthy	Healthy	Healthy	Healthy	Healthy	Healthy	Healthy	'2'!A1	100%
**3**	Healthy	<0.001	100%	Healthy	Healthy	Healthy	Healthy	Healthy	Healthy	Healthy	Healthy	Healthy	Healthy	'3'!A1	100%
**4**	Healthy	0.05	100%	Healthy	Healthy	Healthy	Healthy	Healthy	Healthy	Healthy	Healthy	Healthy	Healthy	'4'!A1	100%
**5**	Healthy	0.004	100%	Healthy	Healthy	Healthy	Healthy	Healthy	Healthy	Healthy	Healthy	Healthy	Healthy	'5'!A1	100%
**6**	Healthy	<0.001	100%	Healthy	Healthy	Healthy	Healthy	Healthy	Healthy	Healthy	Healthy	Healthy	Cancer	'6'!A1	90%
**7**	Cancer	1	100%	Not Certain	Not Certain	Not Certain	Not Certain	Not Certain	Not Certain	Not Certain	Not Certain	Not Certain	Not Certain	'7'!A1	0%
**8**	Cancer	1	100%	Not Certain	Not Certain	Cancer	Not Certain	Not Certain	Cancer	Not Certain	Not Certain	Not Certain	Not Certain	'8'!A1	20%
**9**	Cancer	1	100%	Cancer	Cancer	Cancer	Cancer	Cancer	Cancer	Cancer	Cancer	Cancer	Cancer	'9'!A1	100%
**10**	Cancer	1	100%	Cancer	Cancer	Cancer	Cancer	Cancer	Cancer	Cancer	Cancer	Cancer	Cancer	'10'!A1	100%
**11**	Cancer	1	100%	Cancer	Cancer	Cancer	Cancer	Cancer	Cancer	Cancer	Cancer	Cancer	Not Certain	'11'!A1	90%
**12**	Cancer	1	100%	Not Certain	Cancer	Cancer	Cancer	Cancer	Cancer	Cancer	Not Certain	Cancer	Not Certain	'12'!A1	70%
**13**	Cancer	1	100%	Cancer	Not Certain	Cancer	Not Certain	Not Certain	Cancer	Not Certain	Not Certain	Cancer	Not Certain	'13'!A1	40%
**14**	Cancer	1	100%	Cancer	Cancer	Cancer	Cancer	Cancer	Cancer	Cancer	Cancer	Cancer	Cancer	'14'!A1	100%
**15**	Cancer	1	100%	Not Certain	Not Certain	Cancer	Cancer	Cancer	Cancer	Cancer	Cancer	Cancer	Cancer	'15'!A1	80%
**Years of experience**				**3**	**2**	**20**	**10**	**6**	**33**	**18**	**18**	**7**	**2**	**Average**	**79%**

Intrpretation: 1 = Cancer 100%; and < 0.5 = Healthy

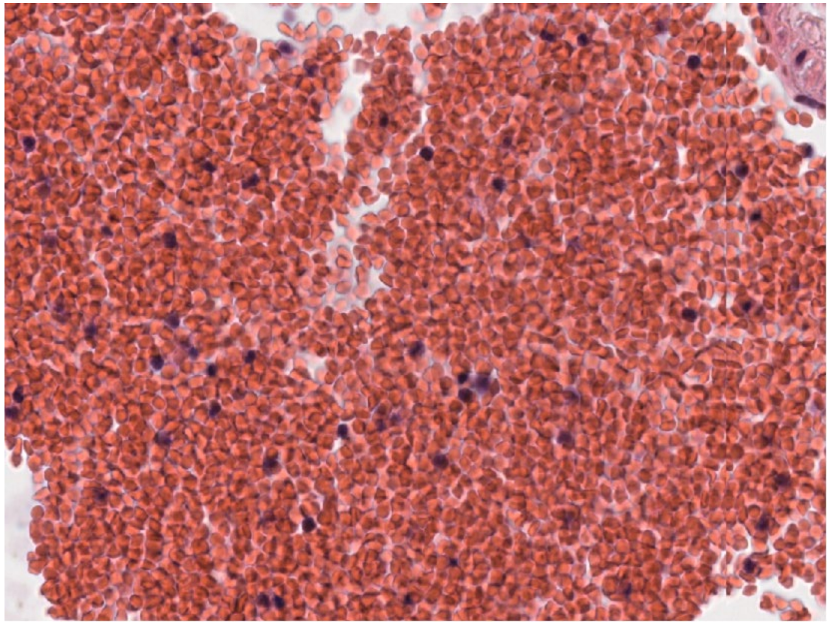

Out[13]: <matplotlib.image.AxesImage at 0x22e9df72790>

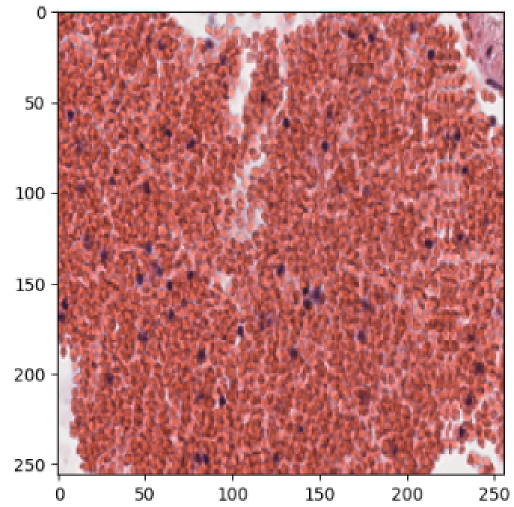

In [14]:
```
# predict the class of the image
# predict method gives a probability whether the slide contains
# malignancy or not
# anything less than 0.5 means no malignancy found,
# greather than or equal to 0.5 means malignancy found
model.predict_(image)
# 1 means 100% cancerous
# 0 means 100% Healthy cell
```

```
1/1 [==============================] - 0s 137ms/step
```

Out[14]: `array([2.6482826e-07], dtype=float32)`

In [15]:
```
# what did the model see to determine that the slide had malignancy
# looking at the top 6 spots where the model found malignancy
malignant_spots(model.model, sample_image_path, confidence = 0.6)
```

```
Creating sliding windows over the picture ...
Getting predictions ...
5/5 [==============================] - 7s 1s/step
No malignant spot was found with the used confidence rate 0.6. Try to lower it a bit!
```

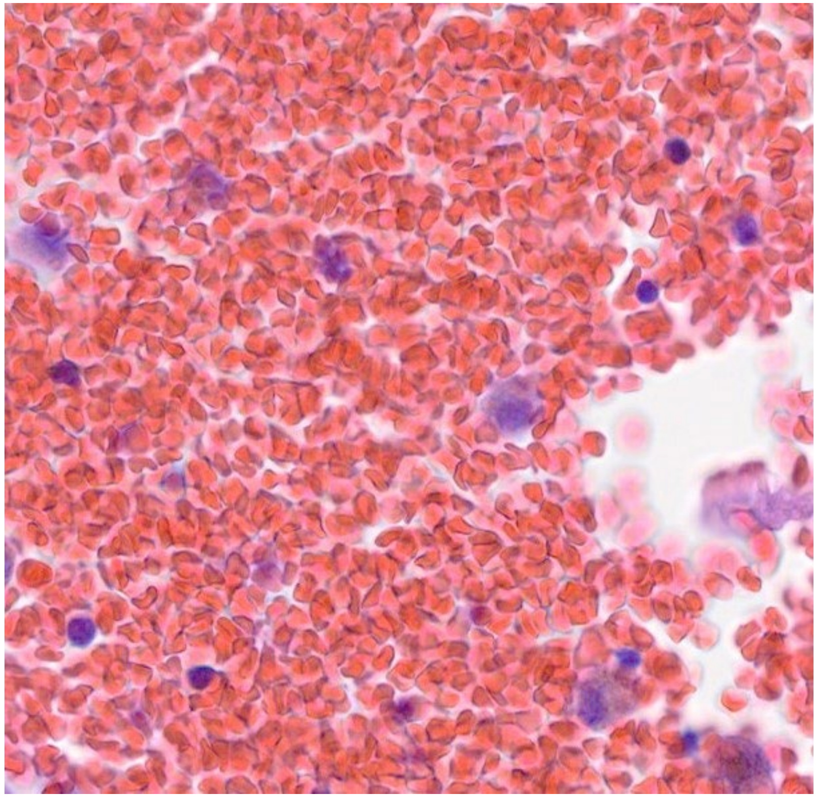

Out[18]: <matplotlib.image.AxesImage at 0x22e9dd4fed0>

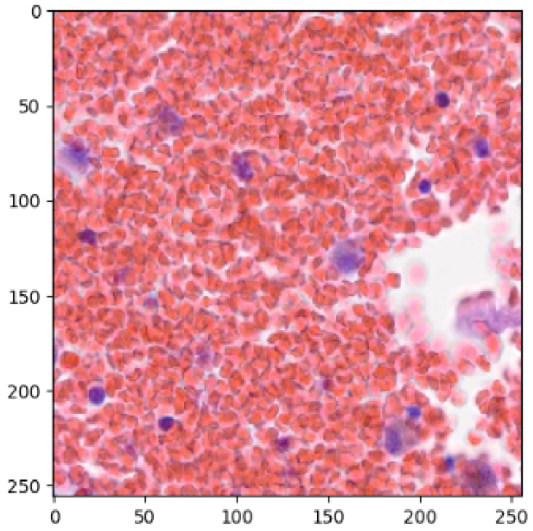

In [19]:
```
# predict the class of the image
# predict method gives a probability whether the slide contains
# malignancy or not
# anything less than 0.5 means no malignancy found,
# greather than or equal to 0.5 means malignancy found
model.predict_(image)
# 1 means 100% cancerous
# 0 means 100% Healthy cell
```

```
1/1 [==============================] - 0s 214ms/step
```

Out[19]: `array([1.4887766e-08], dtype=float32)`

In [15]:
```
# what did the model see to determine that the slide had malignancy
# looking at the top 6 spots where the model found malignancy
malignant_spots(model.model, sample_image_path, confidence = 0.6)
```

```
Creating sliding windows over the picture ...
Getting predictions ...
5/5 [==============================] - 7s 1s/step
No malignant spot was found with the used confidence rate 0.6. Try to lower it a bit!
```

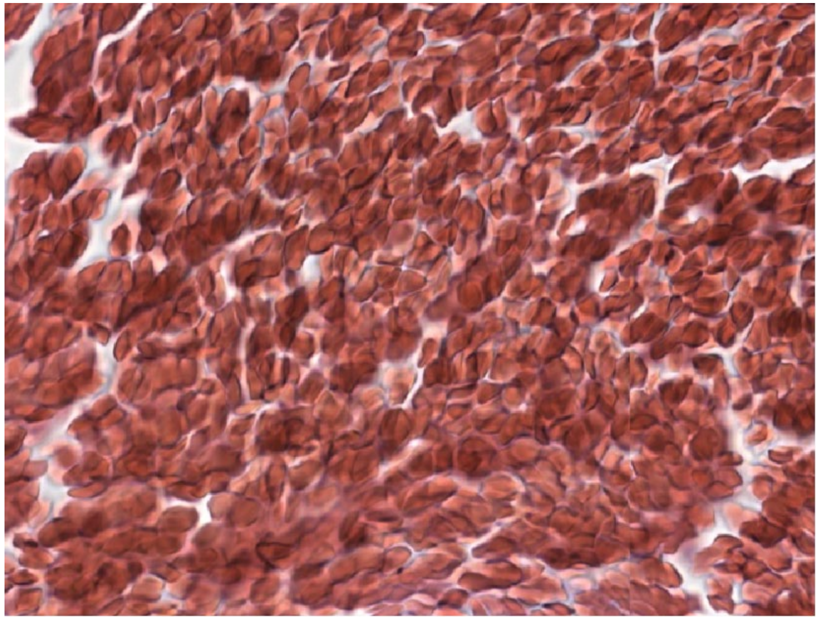

```
Out[22]: <matplotlib.image.AxesImage at 0x22ea66f1290>
```

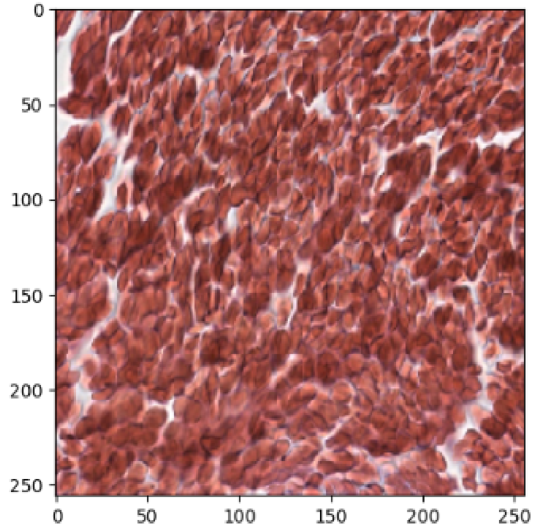


```
In [23]: # predict the class of the image
         # predict method gives a probability whether the slide contains
         # malignancy or not
         # anything less than 0.5 means no malignancy found,
         # greather than or equal to 0.5 means malignancy found
         model.predict_(image)
         # 1 means 100% cancerous
         # 0 means 100% Healthy cell

         1/1 [==============================] - 0s 80ms/step

Out[23]: array([0.00013303], dtype=float32)

In [24]: # what did the model see to determine that the slide had malignancy
         # looking at the top 6 spots where the model found malignancy
         malignant_spots(model.model, sample_image_path, confidence = 0.6)

         Creating sliding windows over the picture ...
         Getting predictions ...
         5/5 [==============================] - 7s 1s/step
         No malignant spot was found with the used confidence rate 0.6. Try to lower it a bit!
```

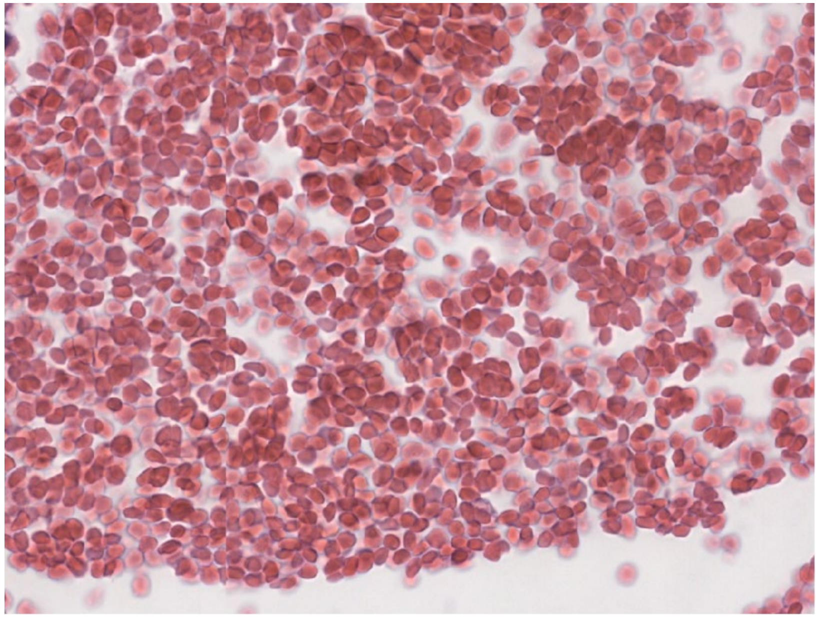

Out[27]: <matplotlib.image.AxesImage at 0x22ea78b0890>

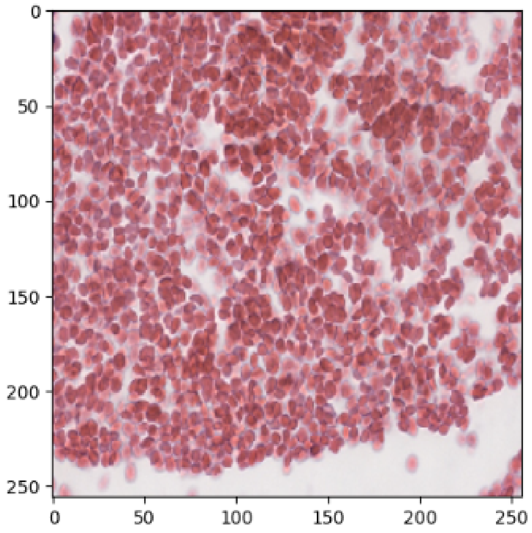

In [28]:
```
# predict the class of the image
# predict method gives a probability whether the slide contains
# malignancy or not
# anything less than 0.5 means no malignancy found,
# greather than or equal to 0.5 means malignancy found
model.predict_(image)
# 1 means 100% cancerous
# 0 means 100% Healthy cell
```

```
1/1 [==============================] - 0s 82ms/step
```

Out[28]:
```
array([0.05565841], dtype=float32)
```

In [29]:
```
# what did the model see to determine that the slide had malignancy
# looking at the top 6 spots where the model found malignancy
malignant_spots(model.model, sample_image_path, confidence = 0.6)
```

```
Creating sliding windows over the picture ...
Getting predictions ...
5/5 [==============================] - 7s 1s/step
No malignant spot was found with the used confidence rate 0.6. Try to lower it a bit!
```

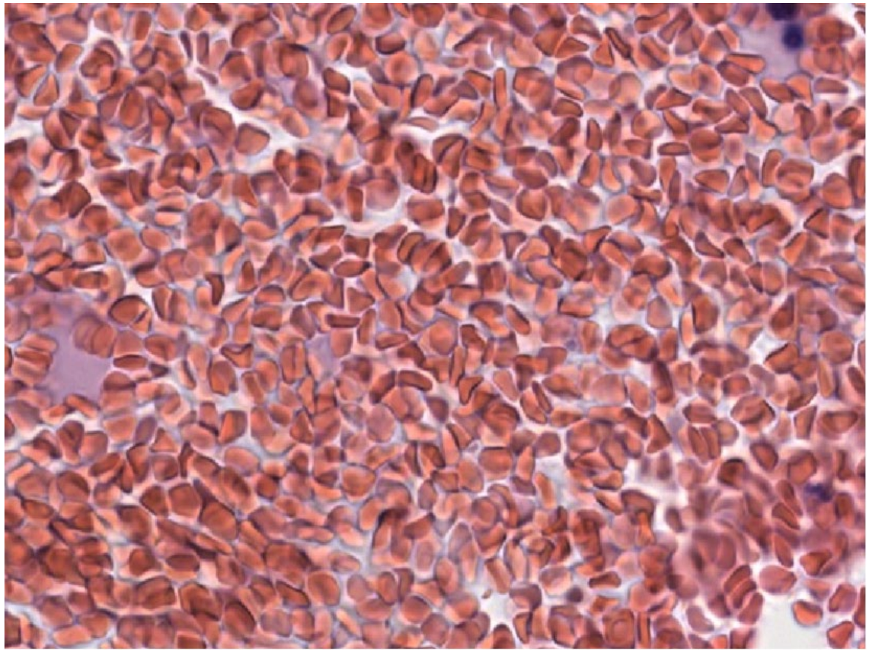

Out[32]: <matplotlib.image.AxesImage at 0x22ea775ec50>

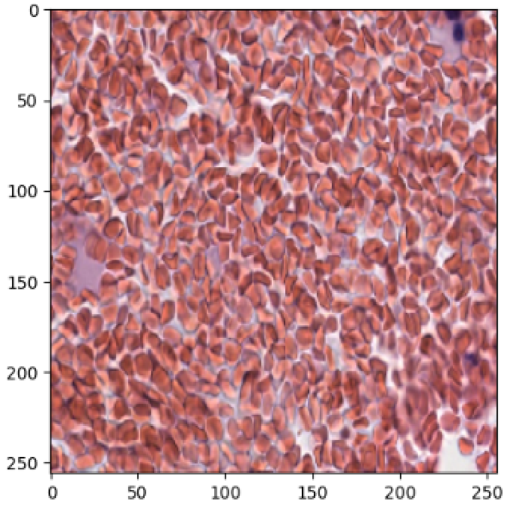

In [33]:
```
# predict the class of the image
# predict method gives a probability whether the slide contains
# malignancy or not
# anything less than 0.5 means no malignancy found,
# greather than or equal to 0.5 means malignancy found
model.predict_(image)
# 1 means 100% cancerous
# 0 means 100% Healthy cell
```

```
1/1 [==============================] - 0s 70ms/step
```

Out[33]:
```
array([0.00417446], dtype=float32)
```

In [34]:
```
# what did the model see to determine that the slide had malignancy
# looking at the top 6 spots where the model found malignancy
malignant_spots(model.model, sample_image_path, confidence = 0.6)
```

```
Creating sliding windows over the picture ...
Getting predictions ...
5/5 [==============================] - 7s 1s/step
No malignant spot was found with the used confidence rate 0.6. Try to lower it a bit!
```

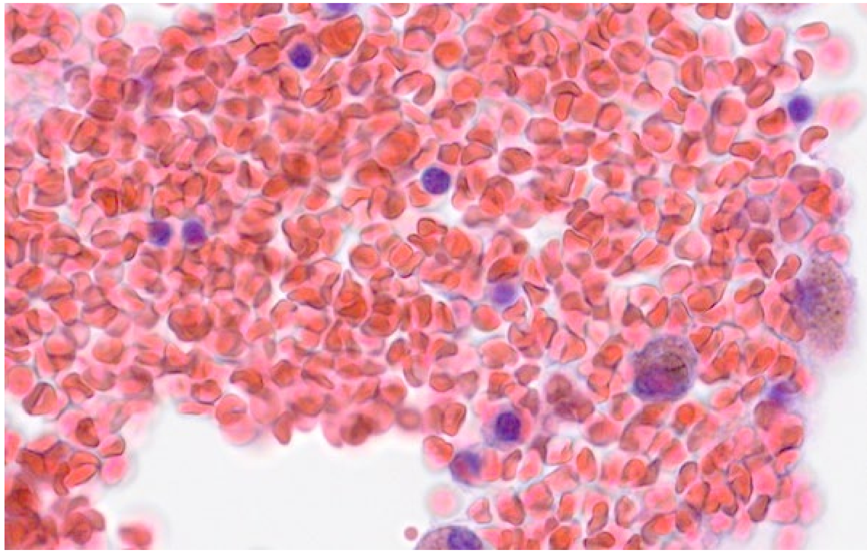

Out[37]: <matplotlib.image.AxesImage at 0x22ea77c1290>

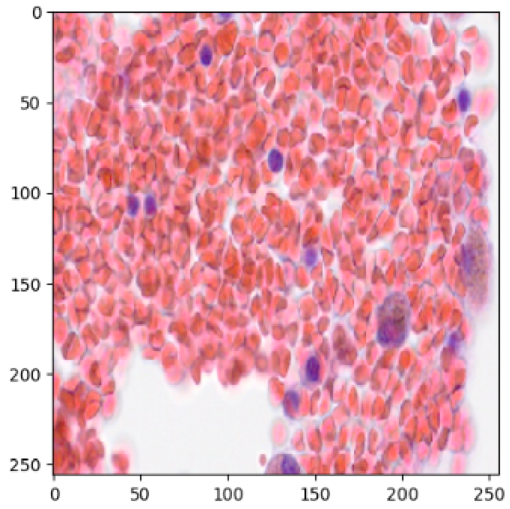

In [38]:
```
# predict the class of the image
# predict method gives a probability whether the slide contains
# malignancy or not
# anything less than 0.5 means no malignancy found,
# greather than or equal to 0.5 means malignancy found
model.predict_(image)
# 1 means 100% cancerous
# 0 means 100% Healthy cell
```

```
1/1 [==============================] - 0s 78ms/step
```

Out[38]: array([7.149009e-07], dtype=float32)

In [39]:
```
# what did the model see to determine that the slide had malignancy
# looking at the top 6 spots where the model found malignancy
malignant_spots(model.model, sample_image_path, confidence = 0.6)
```

```
Creating sliding windows over the picture ...
Getting predictions ...
5/5 [==============================] - 7s 1s/step
No malignant spot was found with the used confidence rate 0.6. Try to lower it a bit!
```

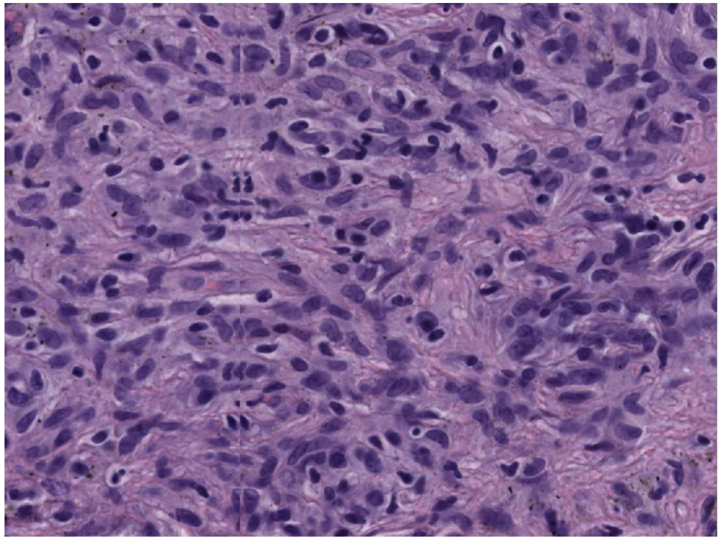

Out[42]: <matplotlib.image.AxesImage at 0x22ea7a12758>

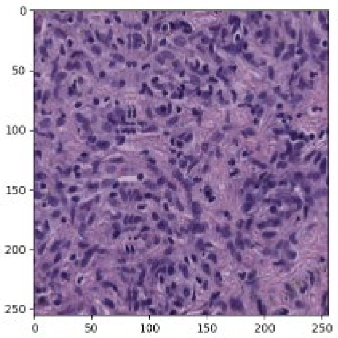

In [43]:

```
# predict the class of the image
# predict method gives a probability whether the slide contains
# malignancy or not
# anything less than 0.5 means no malignancy found,
# greather than or equal to 0.5 means malignancy found
model.predict_(image)
# 1 means 100% cancerous
# 0 means 100% Healthy cell
```

```
1/1 [==============================] - 0s 72ms/step
```

Out[43]: array([1.], dtype=float32)

In [44]:

```
# what did the model see to determine that the slide had malignancy
# looking at the top 6 spots where the model found malignancy
malignant_spots(model.model, sample_image_path, confidence = 0.6)
```

```
Creating sliding windows over the picture ...
Getting predictions ...
5/5 [==============================] - 7s 1s/step
Drawing findings ...
```

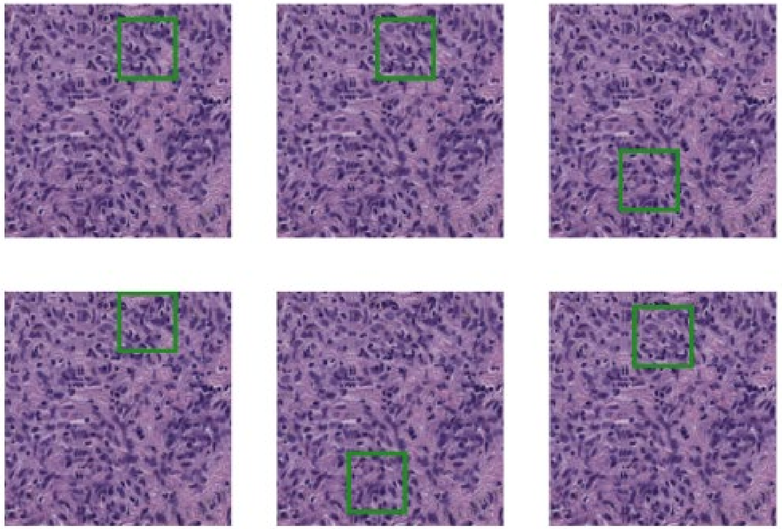

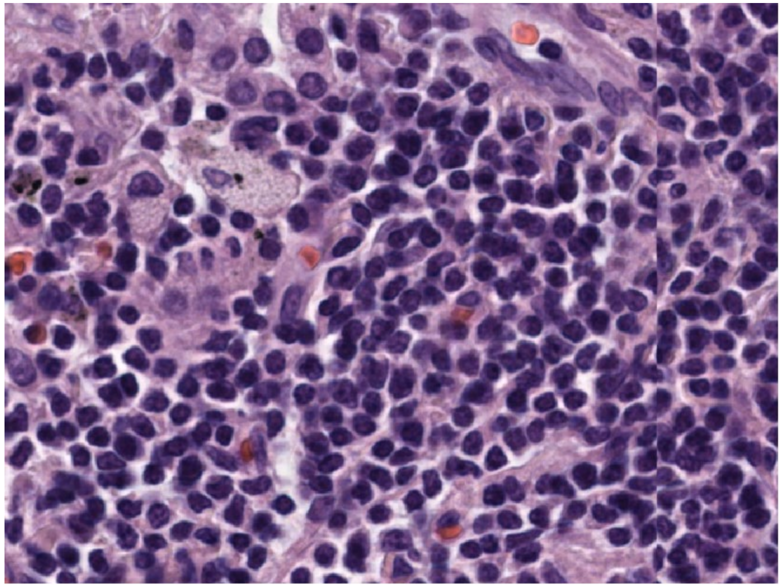

Out[47]: <matplotlib.image.AxesImage at 0x22eb63fb2d0>

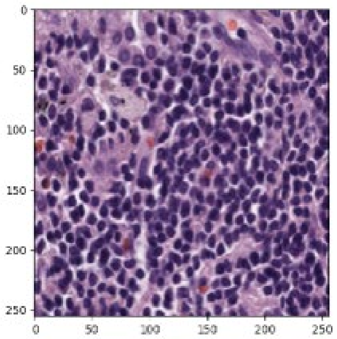

In [48]:
```
# predict the class of the image
# predict method gives a probability whether the slide contains
# malignancy or not
# anything less than 0.5 means no malignancy found,
# greather than or equal to 0.5 means malignancy found
model.predict_(image)
# 1 means 100% cancerous
# 0 means 100% Healthy cell
```

```
1/1 [==============================] - 0s 77ms/step
```

Out[48]: `array([1.], dtype=float32)`

In [49]:
```
# what did the model see to determine that the slide had malignancy
# looking at the top 6 spots where the model found malignancy
malignant_spots(model.model, sample_image_path, confidence = 0.6)
```

```
Creating sliding windows over the picture ...
Getting predictions ...
5/5 [==============================] - 8s 2s/step
Drawing findings ...
```

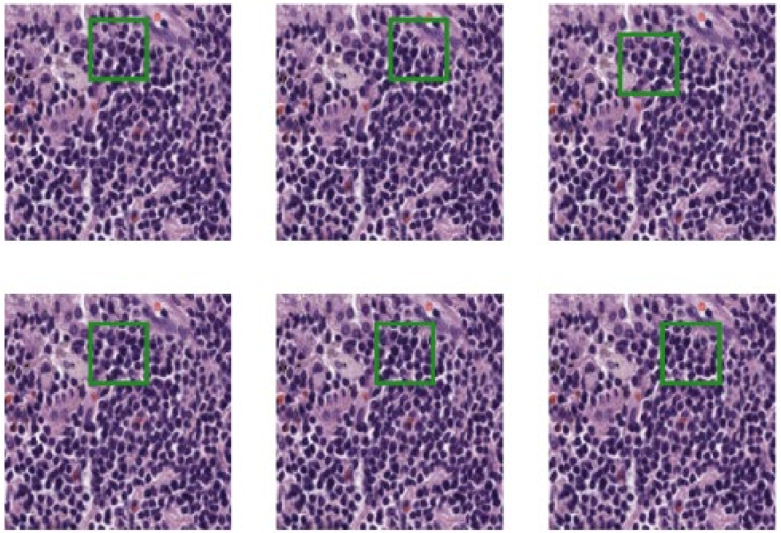

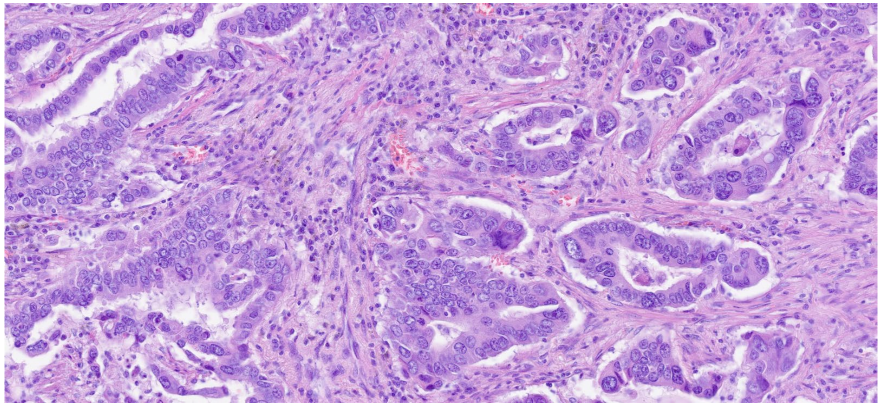

```
Out[52]: <matplotlib.image.AxesImage at 0x22ea8d3ae10>
```

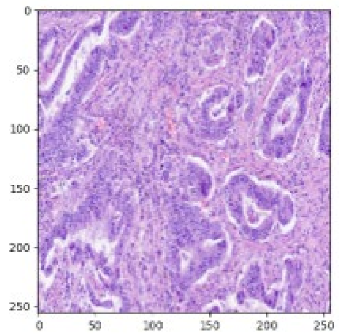


```
In [53]: # predict the class of the image
         # predict method gives a probability whether the slide contains
         # malignancy or not
         # anything less than 0.5 means no malignancy found,
         # greather than or equal to 0.5 means malignancy found
         model.predict_(image)
         # 1 means 100% cancerous
         # 0 means 100% Healthy cell

         1/1 [==============================] - 0s 73ms/step

Out[53]: array([1.], dtype=float32)

In [54]: # what did the model see to determine that the slide had malignancy
         # looking at the top 6 spots where the model found malignancy
         malignant_spots(model.model, sample_image_path, confidence = 0.6)

         Creating sliding windows over the picture ...
         Getting predictions ...
         5/5 [==============================] - 7s 1s/step
         Drawing findings ...
```

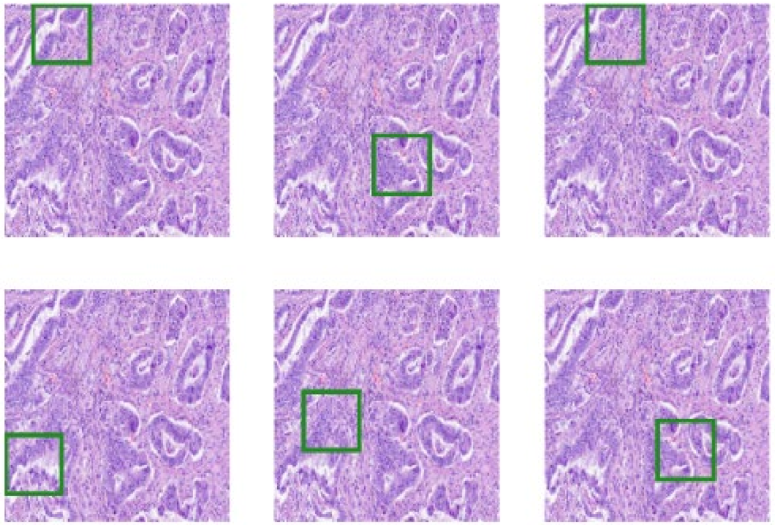

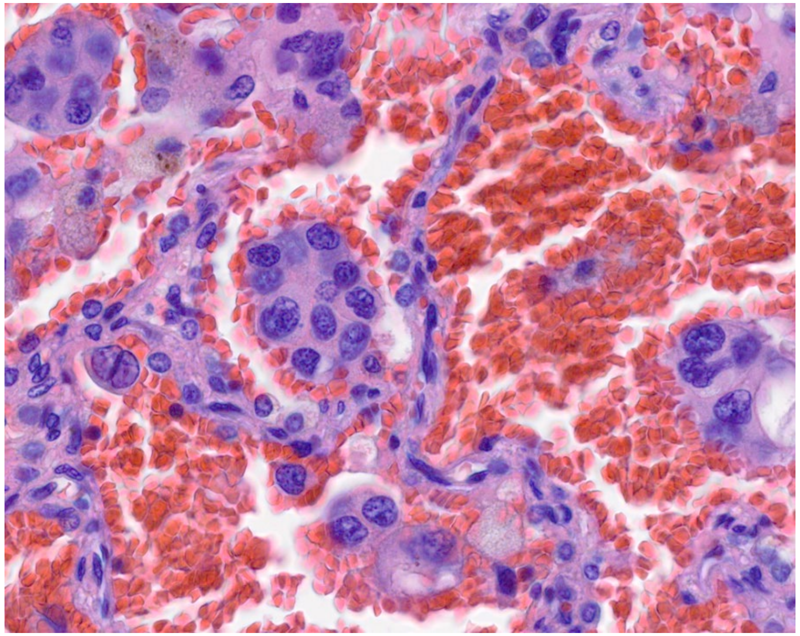

```
Out[57]: <matplotlib.image.AxesImage at 0x22ea8f68250>
```

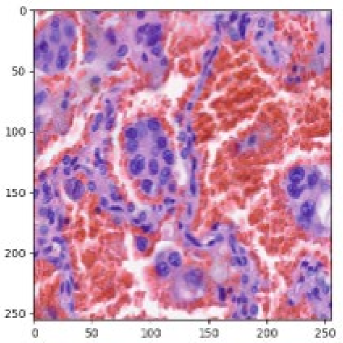


```
In [58]: # predict the class of the image
         # predict method gives a probability whether the slide contains
         # malignancy or not
         # anything less than 0.5 means no malignancy found,
         # greather than or equal to 0.5 means malignancy found
         model.predict_(image)
         # 1 means 100% cancerous
         # 0 means 100% Healthy cell

         1/1 [==============================] - 0s 77ms/step

Out[58]: array([1.], dtype=float32)

In [59]: # what did the model see to determine that the slide had malignancy
         # looking at the top 6 spots where the model found malignancy
         malignant_spots(model.model, sample_image_path, confidence = 0.6)

         Creating sliding windows over the picture ...
         Getting predictions ...
         5/5 [==============================] - 7s 1s/step
         Drawing findings ...
```

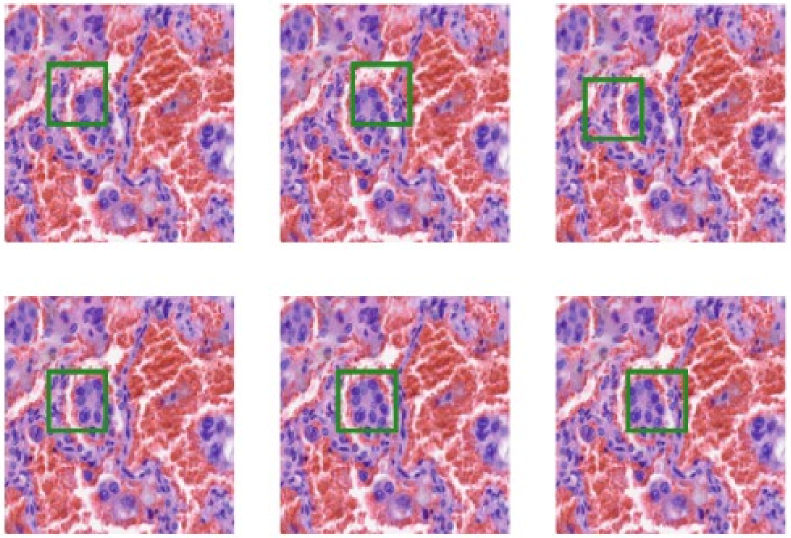

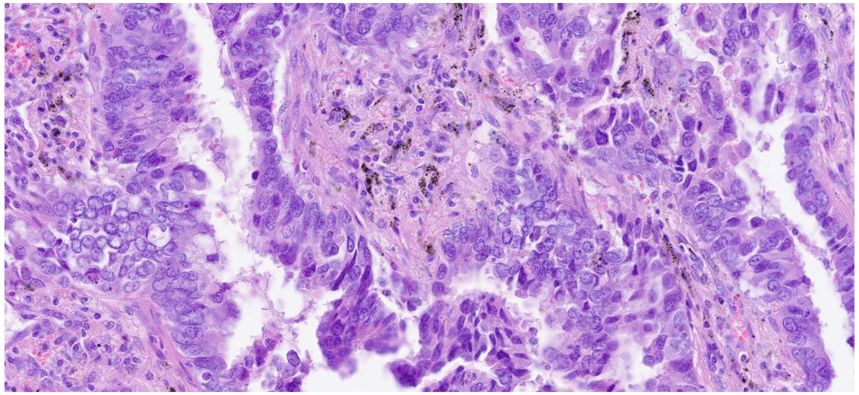

Out[62]: <matplotlib.image.AxesImage at 0x22ea8cb68d0>

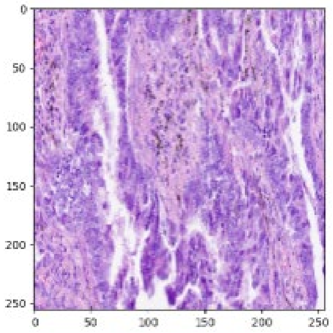

In [63]:
```
# predict the class of the image
# predict method gives a probability whether the slide contains
# malignancy or not
# anything less than 0.5 means no malignancy found,
# greather than or equal to 0.5 means malignancy found
model.predict_(image)
# 1 means 100% cancerous
# 0 means 100% Healthy cell
```

```
1/1 [==============================] - 0s 77ms/step
```

Out[63]: array([1.], dtype=float32)

In [64]:
```
# what did the model see to determine that the slide had malignancy
# looking at the top 6 spots where the model found malignancy
malignant_spots(model.model, sample_image_path, confidence = 0.6)
```

```
Creating sliding windows over the picture ...
Getting predictions ...
5/5 [==============================] - 7s 1s/step
Drawing findings ...
```

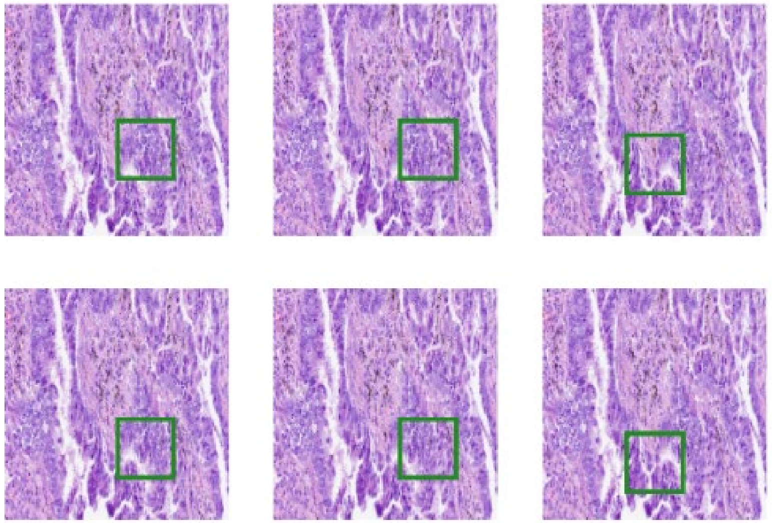

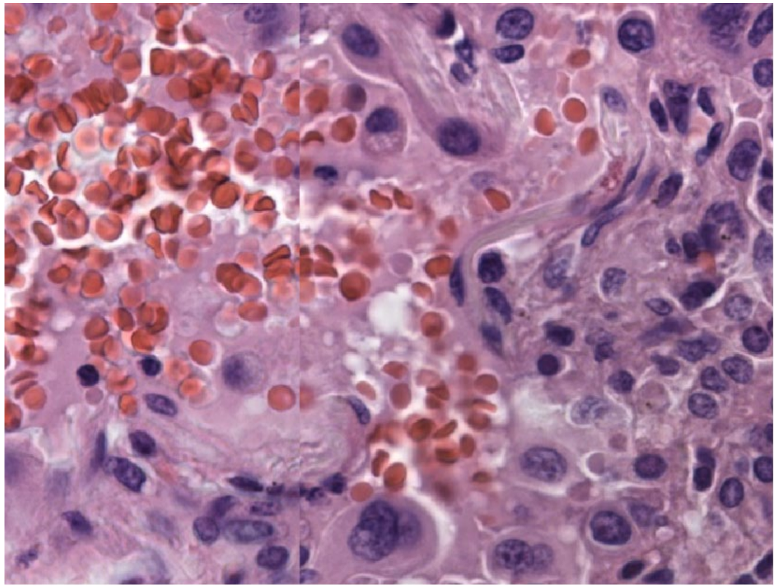

Out[67]: <matplotlib.image.AxesImage at 0x22ea8ac3118>

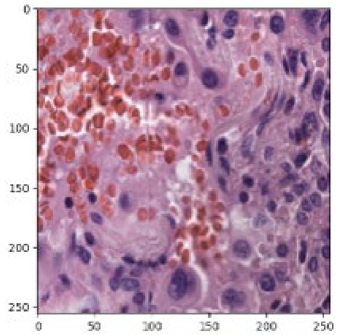

In [68]:
```
# predict the class of the image
# predict method gives a probability whether the slide contains
# malignancy or not
# anything less than 0.5 means no malignancy found,
# greather than or equal to 0.5 means malignancy found
model.predict_(image)
# 1 means 100% cancerous
# 0 means 100% Healthy cell
```

```
1/1 [==============================] - 0s 74ms/step
```

Out[68]: array([1.], dtype=float32)

In [69]:
```
# what did the model see to determine that the slide had malignancy
# looking at the top 6 spots where the model found malignancy
malignant_spots(model.model, sample_image_path, confidence = 0.6)
```

```
Creating sliding windows over the picture ...
Getting predictions ...
5/5 [==============================] - 7s 1s/step
Drawing findings ...
```

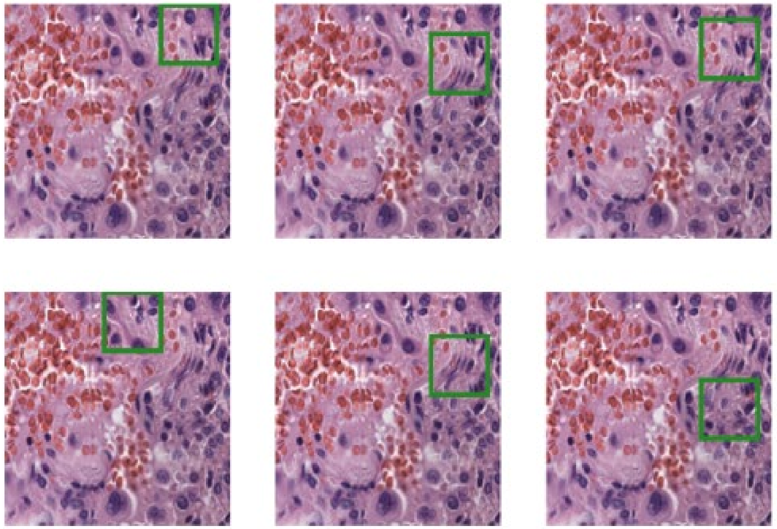

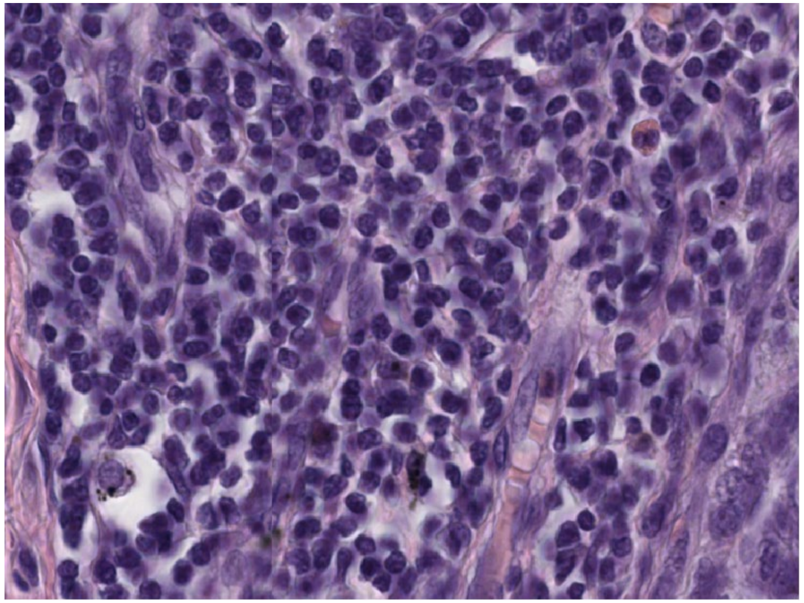

Out[72]: <matplotlib.image.AxesImage at 0x22ea903db18>

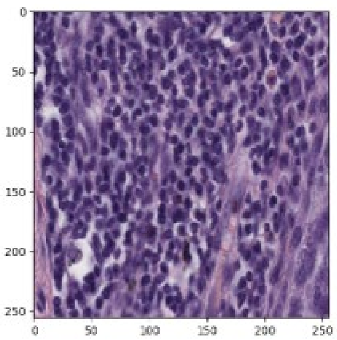

In [73]:
```
# predict the class of the image
# predict method gives a probability whether the slide contains
# malignancy or not
# anything less than 0.5 means no malignancy found,
# greather than or equal to 0.5 means malignancy found
model.predict_(image)
# 1 means 100% cancerous
# 0 means 100% Healthy cell
```

```
1/1 [==============================] - 0s 68ms/step
```

Out[73]: array([1.], dtype=float32)

In [74]:
```
# what did the model see to determine that the slide had malignancy
# looking at the top 6 spots where the model found malignancy
malignant_spots(model.model, sample_image_path, confidence = 0.6)
```

```
Creating sliding windows over the picture ...
Getting predictions ...
5/5 [==============================] - 7s 1s/step
Drawing findings ...
```

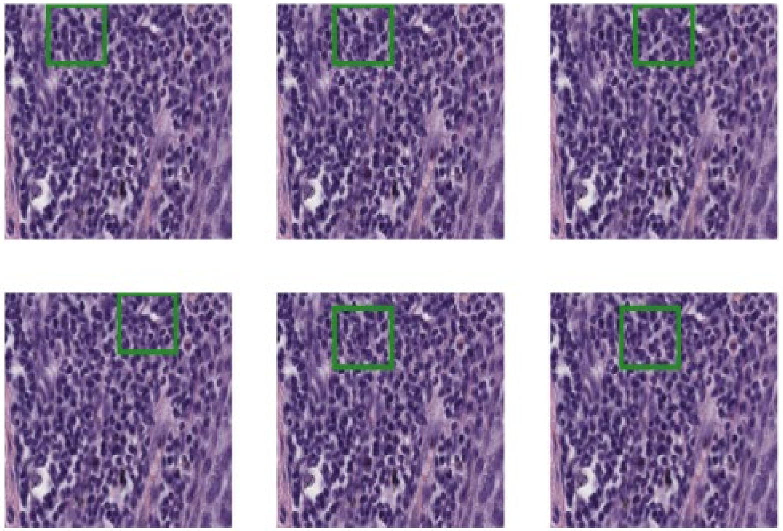

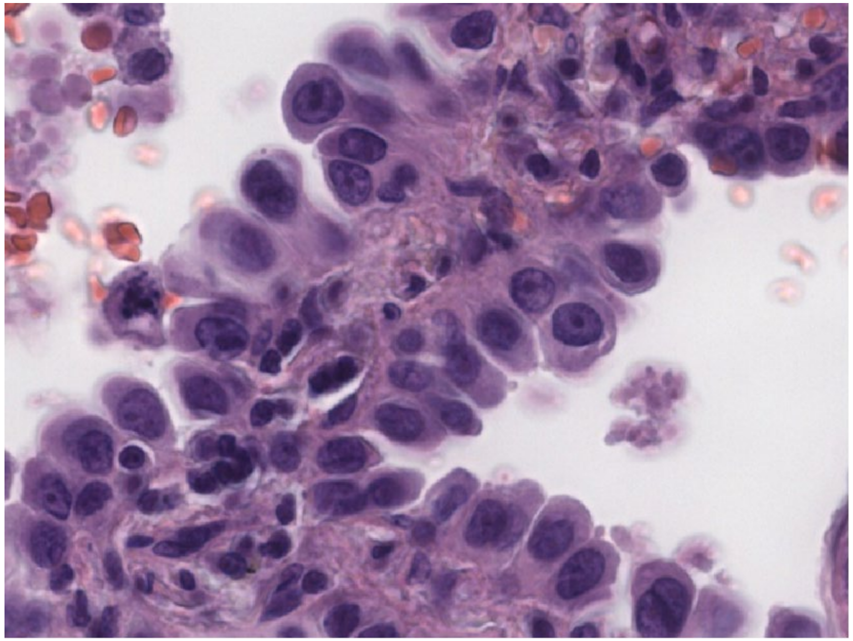

```
Out[77]: <matplotlib.image.AxesImage at 0x22ea9280ad0>
```

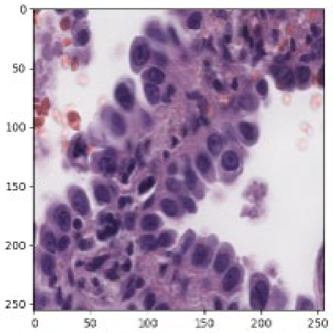


```
In [78]: # predict the class of the image
         # predict method gives a probability whether the slide contains
         # malignancy or not
         # anything less than 0.5 means no malignancy found,
         # greather than or equal to 0.5 means malignancy found
         model.predict_(image)
         # 1 means 100% cancerous
         # 0 means 100% Healthy cell

1/1 [==============================] - 0s 74ms/step

Out[78]: array([1.], dtype=float32)

In [79]: # what did the model see to determine that the slide had malignancy
         # looking at the top 6 spots where the model found malignancy
         malignant_spots(model.model, sample_image_path, confidence = 0.6)

Creating sliding windows over the picture ...
Getting predictions ...
5/5 [==============================] - 7s 1s/step
Drawing findings ...
```

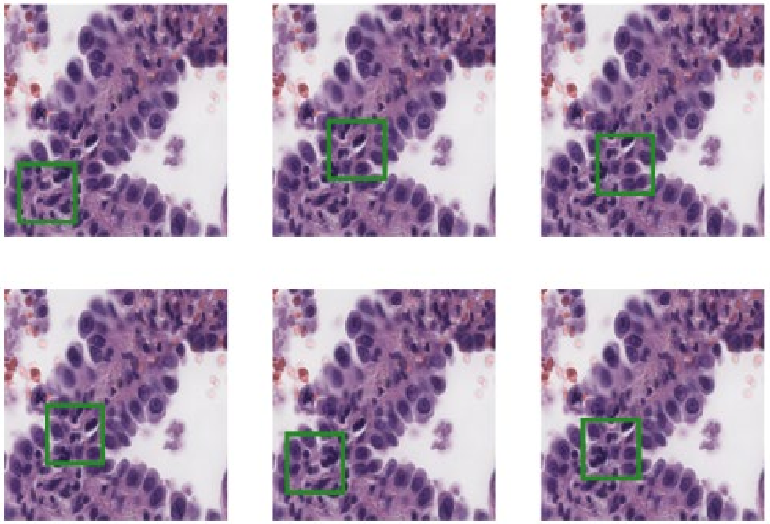

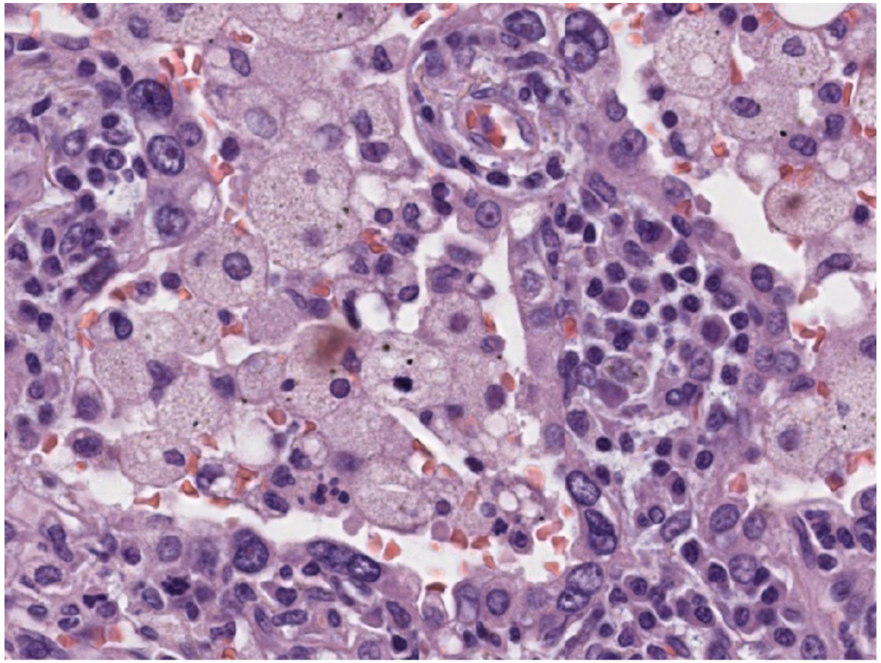

```
Out[82]: <matplotlib.image.AxesImage at 0x22ea9d48110>
```

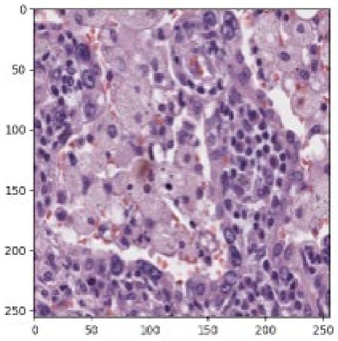


```
In [83]: # predict the class of the image
         # predict method gives a probability whether the slide contains
         # malignancy or not
         # anything less than 0.5 means no malignancy found,
         # greather than or equal to 0.5 means malignancy found
         model.predict_(image)
         # 1 means 100% cancerous
         # 0 means 100% Healthy cell

         1/1 [==============================] - 0s 71ms/step

Out[83]: array([1.], dtype=float32)
```

```
In [84]: # what did the model see to determine that the slide had malignancy
         # looking at the top 6 spots where the model found malignancy
         malignant_spots(model.model, sample_image_path, confidence = 0.6)

         Creating sliding windows over the picture ...
         Getting predictions ...
         5/5 [==============================] - 7s 1s/step
         Drawing findings ...
```

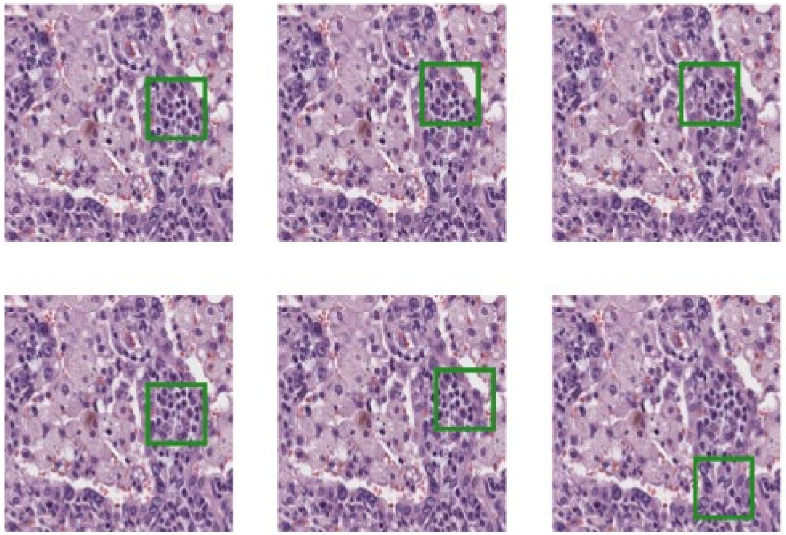

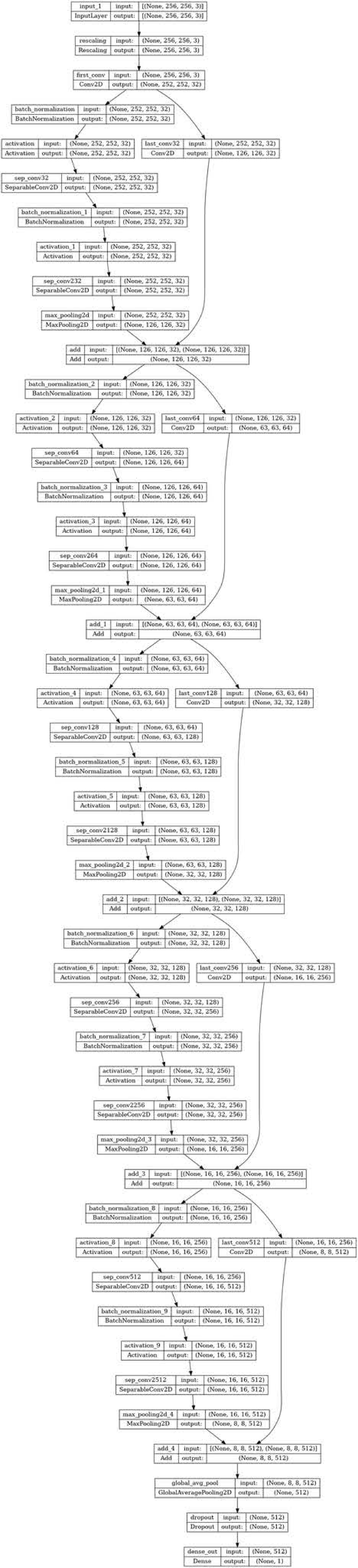

input_1 | input: | [(None, 256, 256, 3)]
InputLayer | output: | [(None, 256, 256, 3)]
rescaling | input: | (None, 256, 256, 3)
Rescaling | output: | (None, 256, 256, 3)
first_conv | input: | (None, 256, 256, 3)
Conv2D | output: | (None, 252, 252, 32)
batch_normalization | input: | (None, 252, 252, 32)
BatchNormalization | output: | (None, 252, 252, 32)
activation | input: | (None, 252, 252, 32)
Activation | output: | (None, 252, 252, 32)
last_conv32 | input: | (None, 252, 252, 32)
Conv2D | output: | (None, 126, 126, 32)
sep_conv32 | input: | (None, 252, 252, 32)
SeparableConv2D | output: | (None, 252, 252, 32)
batch_normalization_1 | input: | (None, 252, 252, 32)
BatchNormalization | output: | (None, 252, 252, 32)
activation_1 | input: | (None, 252, 252, 32)
Activation | output: | (None, 252, 252, 32)
sep_conv232 | input: | (None, 252, 252, 32)
SeparableConv2D | output: | (None, 252, 252, 32)
max_pooling2d | input: | (None, 252, 252, 32)
MaxPooling2D | output: | (None, 126, 126, 32)
add | input: | [(None, 126, 126, 32), (None, 126, 126, 32)]
Add | output: | (None, 126, 126, 32)
batch_normalization_2 | input: | (None, 126, 126, 32)
BatchNormalization | output: | (None, 126, 126, 32)
activation_2 | input: | (None, 126, 126, 32)
Activation | output: | (None, 126, 126, 32)
last_conv64 | input: | (None, 126, 126, 32)
Conv2D | output: | (None, 63, 63, 64)
sep_conv64 | input: | (None, 126, 126, 32)
SeparableConv2D | output: | (None, 126, 126, 64)
batch_normalization_3 | input: | (None, 126, 126, 64)
BatchNormalization | output: | (None, 126, 126, 64)
activation_3 | input: | (None, 126, 126, 64)
Activation | output: | (None, 126, 126, 64)
sep_conv264 | input: | (None, 126, 126, 64)
SeparableConv2D | output: | (None, 126, 126, 64)
max_pooling2d_1 | input: | (None, 126, 126, 64)
MaxPooling2D | output: | (None, 63, 63, 64)
add_1 | input: | [(None, 63, 63, 64), (None, 63, 63, 64)]
Add | output: | (None, 63, 63, 64)
batch_normalization_4 | input: | (None, 63, 63, 64)
BatchNormalization | output: | (None, 63, 63, 64)
activation_4 | input: | (None, 63, 63, 64)
Activation | output: | (None, 63, 63, 64)
last_conv128 | input: | (None, 63, 63, 64)
Conv2D | output: | (None, 32, 32, 128)
sep_conv128 | input: | (None, 63, 63, 64)
SeparableConv2D | output: | (None, 63, 63, 128)
batch_normalization_5 | input: | (None, 63, 63, 128)
BatchNormalization | output: | (None, 63, 63, 128)
activation_5 | input: | (None, 63, 63, 128)
Activation | output: | (None, 63, 63, 128)
sep_conv2128 | input: | (None, 63, 63, 128)
SeparableConv2D | output: | (None, 63, 63, 128)
max_pooling2d_2 | input: | (None, 63, 63, 128)
MaxPooling2D | output: | (None, 32, 32, 128)
add_2 | input: | [(None, 32, 32, 128), (None, 32, 32, 128)]
Add | output: | (None, 32, 32, 128)
batch_normalization_6 | input: | (None, 32, 32, 128)
BatchNormalization | output: | (None, 32, 32, 128)
activation_6 | input: | (None, 32, 32, 128)
Activation | output: | (None, 32, 32, 128)
last_conv256 | input: | (None, 32, 32, 128)
Conv2D | output: | (None, 16, 16, 256)
sep_conv256 | input: | (None, 32, 32, 128)
SeparableConv2D | output: | (None, 32, 32, 256)
batch_normalization_7 | input: | (None, 32, 32, 256)
BatchNormalization | output: | (None, 32, 32, 256)
activation_7 | input: | (None, 32, 32, 256)
Activation | output: | (None, 32, 32, 256)
sep_conv2256 | input: | (None, 32, 32, 256)
SeparableConv2D | output: | (None, 32, 32, 256)
max_pooling2d_3 | input: | (None, 32, 32, 256)
MaxPooling2D | output: | (None, 16, 16, 256)
add_3 | input: | [(None, 16, 16, 256), (None, 16, 16, 256)]
Add | output: | (None, 16, 16, 256)
batch_normalization_8 | input: | (None, 16, 16, 256)
BatchNormalization | output: | (None, 16, 16, 256)
activation_8 | input: | (None, 16, 16, 256)
Activation | output: | (None, 16, 16, 256)
last_conv512 | input: | (None, 16, 16, 256)
Conv2D | output: | (None, 8, 8, 512)
sep_conv512 | input: | (None, 16, 16, 256)
SeparableConv2D | output: | (None, 16, 16, 512)
batch_normalization_9 | input: | (None, 16, 16, 512)
BatchNormalization | output: | (None, 16, 16, 512)
activation_9 | input: | (None, 16, 16, 512)
Activation | output: | (None, 16, 16, 512)
sep_conv2512 | input: | (None, 16, 16, 512)
SeparableConv2D | output: | (None, 16, 16, 512)
max_pooling2d_4 | input: | (None, 16, 16, 512)
MaxPooling2D | output: | (None, 8, 8, 512)
add_4 | input: | [(None, 8, 8, 512), (None, 8, 8, 512)]
Add | output: | (None, 8, 8, 512)
global_avg_pool | input: | (None, 8, 8, 512)
GlobalAveragePooling2D | output: | (None, 512)
dropout | input: | (None, 512)
Dropout | output: | (None, 512)
dense_out | input: | (None, 512)
Dense | output: | (None, 1)

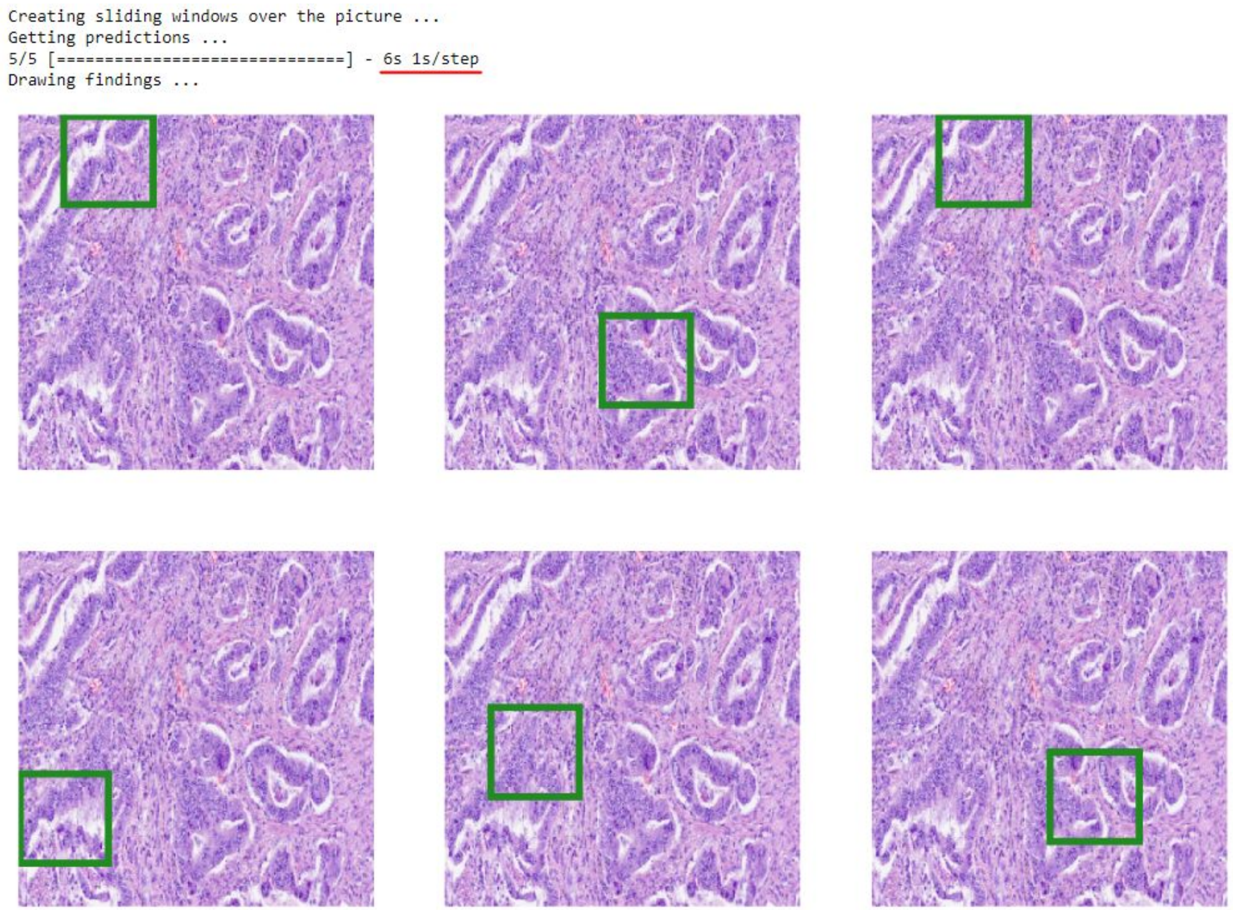

Image from our collected slides after running through the model

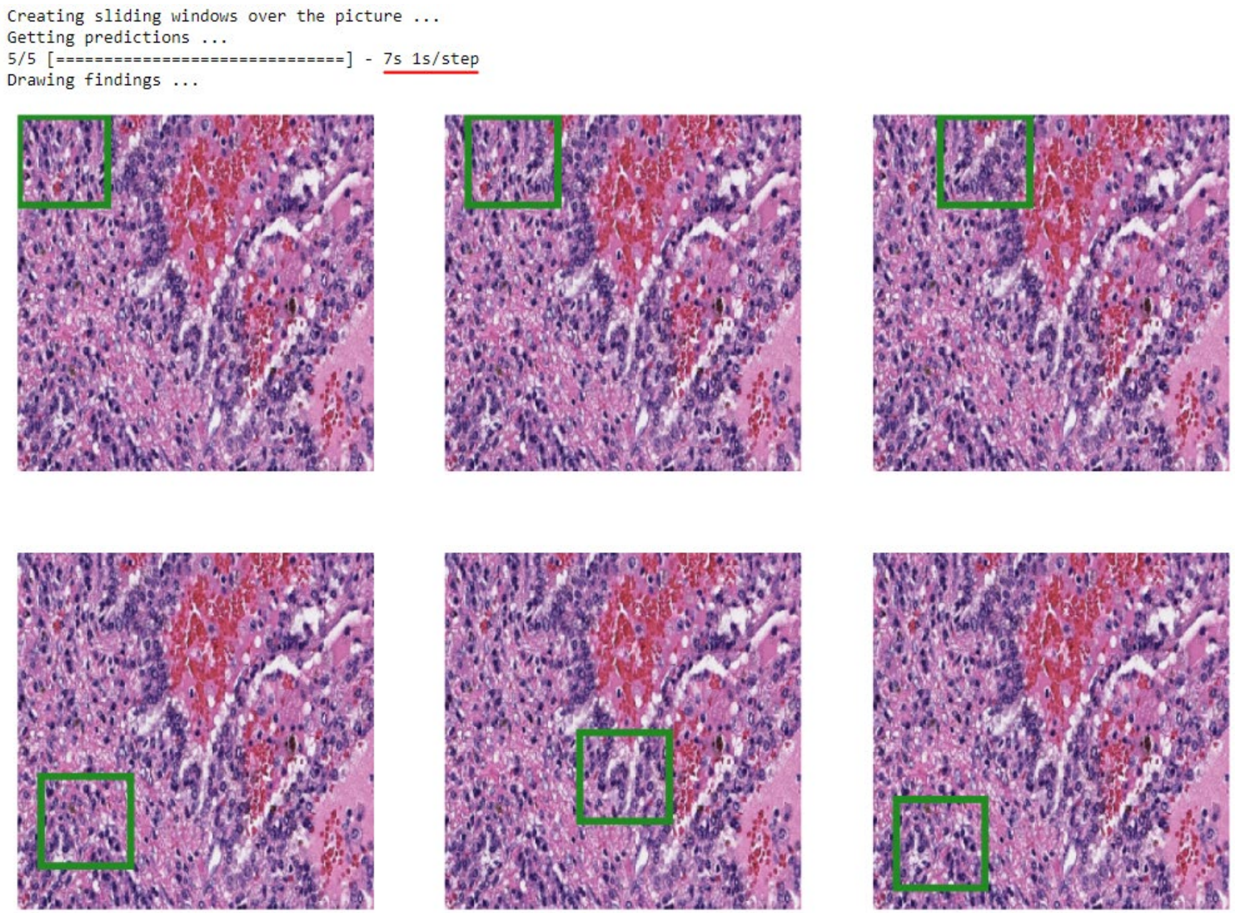

Image from external source after running through the model

Table S1. Comparing the results of the model by images from Department of Pathology and Laboratory Medicine at Dartmouth-Hitchcock Medical Center (DHMC) [1].

Image nr	Dartmouth-Hitchcock MC	Our model
**1**	solid	1
**2**	solid	1
**3**	solid	1
**4**	solid	1
**5**	lepidic	1
**6**	acinar	1
**7**	acinar	1
**8**	solid	1
**9**	acinar	1
**10**	acinar	1
**11**	acinar	1
**12**	solid	1
**13**	solid	1
**14**	acinar	1
**15**	acinar	1
**16**	acinar	1
**17**	solid	1
**18**	lepidic	1
**19**	lepidic	1
**20**	micropapillary	1
**21**	solid	1
**22**	solid	1
**23**	acinar	1
**24**	papillary	1
**25**	solid	1
**26**	acinar	1
**27**	acinar	1
**28**	solid	1
**29**	solid	1
**30**	solid	1
**31**	micropapillary	1
**32**	micropapillary	1
**33**	lepidic	1
**34**	acinar	1
**35**	lepidic	1
**36**	acinar	1
**37**	solid	1
**38**	lepidic	1

**39**	solid	1
**40**	solid	1
**41**	solid	1
**42**	acinar	1
**43**	solid	1
**44**	solid	1
**45**	solid	1
**46**	solid	1
**47**	solid	1
**48**	acinar	1
**49**	micropapillary	1
**50**	acinar	1
**51**	papillary	1
**52**	lepidic	1
**53**	micropapillary	1
**54**	acinar	1
**55**	solid	1
**56**	acinar	1
**57**	acinar	1
**58**	acinar	1
**59**	acinar	1
**60**	micropapillary	1
**61**	acinar	1
**62**	solid	1
**63**	lepidic	1
**64**	acinar	1
**65**	solid	1
**66**	acinar	1
**67**	acinar	1
**68**	acinar	1
**69**	solid	1
**70**	solid	1
**71**	solid	1
**72**	solid	1
**73**	solid	1
**74**	acinar	1
**75**	solid	1
**76**	solid	1
**77**	solid	1
**78**	acinar	1
**79**	acinar	1
**80**	acinar	1
**81**	lepidic	1

**82**	acinar	1
**83**	acinar	1
**84**	acinar	1
**85**	acinar	1
**86**	lepidic	1
**87**	lepidic	1
**88**	acinar	1
**89**	acinar	1
**90**	acinar	1
**91**	solid	1
**92**	acinar	1
**93**	lepidic	1
**94**	acinar	1
**95**	solid	1
**96**	papillary	1
**97**	acinar	1
**98**	acinar	1
**99**	acinar	1
**100**	acinar	1
**101**	lepidic	1
**102**	lepidic	1
**103**	acinar	1
**104**	acinar	1
**105**	acinar	1

Refrences

1. Wei, J.W.; Tafe, L.J.; Linnik, Y.A.; Vaickus, L.J.; Tomita, N.; Hassanpour, S. Pathologist-Level Classification of Histologic Patterns on Resected Lung Adenocarcinoma Slides with Deep Neural Networks. *Sci Rep* **2019**, *9*, 3358, doi:10.1038/s41598-019-40041-7.
